# Enquiring About Tolerance (EAT) study – feasibility of early introduction of allergenic foods and impact on breastfeeding

**DOI:** 10.1186/2045-7022-5-S3-O6

**Published:** 2015-03-30

**Authors:** Michael Perkin, Kirsty Logan, Joanna Craven, Tom Marrs, Suzana Radulovic, Carsten Flohr, Gideon Lack

**Affiliations:** 1 Children's Allergies Department, King’s College London, London, UK; 2 St John's Institute of Dermatology, London, UK

## Background

The introduction of multiple allergenic foods in early infancy has not been attempted in a randomized controlled trial setting and the impact on breastfeeding performance is unknown.

## Method

1,303 exclusively breastfeeding (EBF) infants were recruited to the EAT Study at 3 months of age and randomized to either EBF until around 6 months of age (Standard Introduction Group - SIG) or early introduction of 6 allergenic foods (cow's milk, egg, fish, wheat, sesame and peanut) alongside breastfeeding (Early Introduction Group - EIG). Data from the UK 2010 Infant Feeding Survey (IFS) was used to compare EAT Study data to UK population data.

## Results

Mothers in the SIG were significantly more likely, and mothers in the EIG significantly less likely to be EBF than IFS equivalent mothers at 4, 5 and 6 months of age (p< 0.0005). Any breastfeeding rates in the two groups were very similar and significantly better than 2010 IFS equivalent mothers at 4, 5 and 6 months of age (p<0.0005).

**Figure 1:**
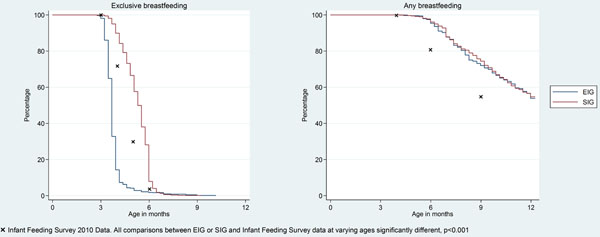


Overall per protocol compliance in the SIG was high. In the EIG, cow's milk proved to be the easiest food to introduce while sesame and egg had the slowest pace of introduction. Non-consumption rates were very low for all foods. By 5 months the median consumption frequency in the EIG was 2-3 times a week for all 6 foods with minimal consumption in the SIG. Comparisons between groups for each food at every time point were statistically significant (p< 0.0005).

## Conclusion

The EAT study is confirmation that the introduction of 6 of the principal allergenic foods in early infancy is achievable and without any deleterious effect on breastfeeding rates.

